# The Case for N-acetylcysteine in Dengue Associated Liver Injury: Time for Definitive Evidence

**DOI:** 10.1093/ofid/ofag421

**Published:** 2026-07-07

**Authors:** Angela McBride, Nguyen Lam Vuong, Sudeep Adhikari, Su Myat Han, Po Ying Chia, Huynh Trung Trieu, Phung Khanh Lam, Ho Quang Chanh, Giri Rajahram, Peter Horby, Tsin Wen Yeo, Evelyne Kestelyn, James Watson, Truong Ngoc Trung, Sophie Yacoub

**Affiliations:** Pandemic Sciences Institute, University of Oxford, Oxford, UK; Oxford University Clinical Research Unit, Ho Chi Minh City, Vietnam; Department of Medical Statistics and Informatics, University of Medicine and Pharmacy at Ho Chi Minh City, Ho Chi Minh City, Vietnam; Oxford University Clinical Research Unit Nepal, Lalitpur, Nepal; School of Tropical Medicine and Global Health, Nagasaki University, Nagasaki, Japan; National Centre for Infectious Diseases, Singapore, Singapore; Department of Infectious Diseases, Tan Tok Seng Hospital, Singapore, Singapore; Hospital for Tropical Diseases, Ho Chi Minh City, Vietnam; Saw Swee Hock School of Public Health, National University of Singapore, Singapore, Singapore; Oxford University Clinical Research Unit, Ho Chi Minh City, Vietnam; Department of Medicine, Queen Elizabeth II Hospital, Kota Kinabalu, Malaysia; Clinical Research Unit, Kota Kinabalu Sabah-Menzies School of Health Research, Kota Kinabalu, Malaysia; Pandemic Sciences Institute, University of Oxford, Oxford, UK; National Centre for Infectious Diseases, Singapore, Singapore; Department of Infectious Diseases, Tan Tok Seng Hospital, Singapore, Singapore; Pandemic Sciences Institute, University of Oxford, Oxford, UK; Oxford University Clinical Research Unit, Ho Chi Minh City, Vietnam; Pandemic Sciences Institute, University of Oxford, Oxford, UK; Hospital for Tropical Diseases, Ho Chi Minh City, Vietnam; Pandemic Sciences Institute, University of Oxford, Oxford, UK; Oxford University Clinical Research Unit, Ho Chi Minh City, Vietnam

**Keywords:** acute liver failure, dengue, host-directed therapy, liver injury, N-acetylcysteine

## Abstract

Dengue-associated liver injury is common, prognostically significant, and associated with high mortality at severe thresholds. Oxidative stress is a key, potentially modifiable mechanism. N-acetylcysteine has strong biological plausibility and supportive preclinical data, but clinical evidence remains limited, underscoring the need for well-designed randomized trials to inform practice and policy.

## BURDEN AND MECHANISMS OF DENGUE-ASSOCIATED LIVER INJURY

Dengue virus infection is now endemic in more than 100 countries worldwide, driven by climate change, urbanization, and population mobility, and causes an estimated 105 million symptomatic infections annually [1]. Despite this substantial and growing burden, there are no proven therapies to prevent progression to severe disease or death. While antiviral development continues, increasing attention has focused on interventions that modify the host response to infection.

Liver involvement is a near-universal feature of symptomatic dengue infection. In a large dataset pooled from 2555 hospitalized patients enrolled in clinical trials and prospective cohort studies in Vietnam, over 95% of adults with dengue had elevated transaminases; 12% developed moderate liver injury (AST or ALT >400 IU/L), and 2% developed severe liver injury (>1000 IU/L) [2–6]. Importantly, these thresholds were associated with mortality: 3.1% and 14.6%, respectively, compared with 0.2% in those with transaminases <400 IU/L (unpublished data). Similar findings have been observed across endemic settings, and a recent meta-analysis reported a pooled mortality of 47% among patients with dengue-associated acute liver failure (ALF) [7].

The mechanisms underlying liver injury in dengue are multifactorial and incompletely understood. Proposed contributors include oxidative stress and glutathione depletion, immune-mediated hepatocyte injury, hypoxic or ischemic damage related to plasma leakage and shock, and direct viral cytotoxicity [8, 9]. Among these, oxidative stress represents a potentially modifiable pathway. Experimental and clinical data suggest that dengue infection induces a pro-oxidant state, rendering hepatocytes more vulnerable to secondary insults and impairing cellular recovery [10, 11].

Taken together, dengue-associated liver injury is common, prognostically significant, and biologically heterogeneous, but characterized by pathways that are potentially amenable to targeted host-directed therapy.

## BIOLOGICAL RATIONALE FOR N-ACETYLCYSTEINE IN DENGUE-ASSOCIATED LIVER INJURY

N-acetylcysteine (NAC) is a precursor of cysteine, the rate-limiting substrate for intracellular glutathione synthesis. Glutathione is the primary cellular antioxidant responsible for maintaining redox homeostasis and protecting hepatocytes from oxidative injury. In addition, glutathione modulates inflammatory signaling pathways, including inhibition of nuclear factor kappa B (NF-κB), thereby attenuating downstream proinflammatory gene transcription [12].

In the context of dengue infection, limited in vitro and animal data suggest that dengue virus induces a glutathione-deficient state, leading to increased oxidative stress and hepatocyte vulnerability [13]. Restoration of glutathione stores through NAC administration therefore represents a biologically plausible strategy to mitigate liver injury. Beyond its antioxidant effects, NAC has also been shown to exert vasodilatory and inotropic properties, which may improve microcirculatory perfusion and tissue oxygenation during shock—a key determinant of hepatic injury in severe dengue [14]

An additional and clinically important consideration is the widespread use of paracetamol in dengue. Paracetamol is the only analgesic and antipyretic recommended by the World Health Organization (WHO) for dengue and is commonly administered at doses of up to 4 g/day [15]. A double-blind, randomized controlled trial in Thailand reported a higher frequency of transaminase elevations among adults with dengue receiving standard-dose paracetamol compared with placebo, despite a median daily dose of ∼1.5 g/day [16]. However, the study was not designed to establish causality, no cases of liver failure were observed, and hepatic enzyme elevations are common in dengue irrespective of drug exposure. As such, the clinical significance of these findings remains uncertain. It has been hypothesized that acute dengue infection may be associated with reduced hepatic glutathione reserves, which could plausibly increase susceptibility to paracetamol-related hepatotoxic effects, although this mechanism has not been conclusively demonstrated.

In this context, NAC may serve a potential dual role: attenuating dengue-associated oxidative liver injury while possibly mitigating vulnerability to paracetamol-related hepatotoxicity. Further mechanistic and clinical studies are required to clarify these interactions and their implications for routine dengue management.

## CURRENT EVIDENCE FOR NAC IN DENGUE-ASSOCIATED LIVER INJURY

Preclinical studies provide supportive evidence for a protective role of NAC in dengue-associated liver injury. In vitro experiments using HepG2 cells infected with dengue virus serotype 2 demonstrated dose-dependent restoration of cell viability following NAC administration [11]. In murine models, NAC treatment attenuated dengue-induced leucopenia and thrombocytopenia, reduced elevations in AST and ALT, improved hepatic histopathology, and decreased markers of apoptosis and oxidative stress, including restoration of superoxide dismutase and catalase activity [11, 16].

However, human data remain very limited. Evidence consists primarily of case reports, small case series, and retrospective cohort studies [11, 17–20]. Most reports describe improvement in liver enzymes and synthetic function following NAC administration; however, these outcomes are difficult to interpret in the absence of comparator groups and given the expected spontaneous recovery in most dengue patients. Where comparator populations were included, NAC was often administered as part of a broader bundle of supportive care, precluding attribution of benefit to NAC alone [20].

Two retrospective cohort studies reported nonstatistically significant reductions in mortality among patients treated with NAC compared with those who did not receive NAC, but neither study was adequately powered to detect differences in clinical outcomes [17, 18]]. Additional limitations across studies include heterogeneity in NAC dosing and timing, indication bias (with sicker patients more likely to receive NAC), and confounding by intensive supportive care.

Thus, despite encouraging signals from preclinical models and uncontrolled clinical observations, there is currently no high-quality evidence demonstrating that NAC improves clinically meaningful outcomes in dengue-associated liver injury.

## GUIDELINES, POLICY CONTEXT, AND EVIDENCE GAP

Randomized controlled trials in nonparacetamol-related ALF of noninfectious etiology have demonstrated that NAC improves transplant-free survival, particularly when administered early. On this basis, both the American Association for the Study of Liver Diseases and the European Association for the Study of the Liver recommend NAC as standard of care for nonparacetamol ALF. However, these recommendations are largely derived from studies conducted in high-income settings and do not specifically address infectious causes of ALF, which differ substantially in pathophysiology, resource availability and patient populations.

To date, no randomized trials have evaluated NAC in ALF of infectious etiology. In 2021, a proposal to expand the WHO Essential Medicines indication for NAC to include nonparacetamol-related ALF, including dengue, was rejected due to insufficient evidence [21]. Notably, the WHO expert committee concluded that higher-quality studies would be feasible and valuable to inform future consideration [22]. Despite the absence of clinical trial evidence, NAC has subsequently been recommended for yellow fever associated liver injury in the WHO guidelines for clinical management of arboviral diseases [15].

This divergence between guideline recommendations and the available evidence highlights a critical gap. NAC is currently used in routine clinical practice for dengue-associated liver injury in several endemic countries, and suggested dosing regimens are included in national dengue guidelines for Vietnam and Malaysia. However, its efficacy has never been rigorously tested in the populations and settings where dengue is most prevalent. Generating high-quality evidence in these contexts is therefore essential to ensure that current practice is either validated or revised.

## CONCLUSION

Dengue-associated liver injury is common, prognostically important, and associated with substantial mortality at higher degrees of severity. There is strong biological plausibility supporting the use of NAC in this context, including mitigation of oxidative stress, modulation of inflammatory pathways, improvement in microcirculatory perfusion, and attenuation of paracetamol-related hepatotoxicity. NAC is inexpensive, widely available, and already used in dengue patients despite the absence of robust evidence. Given the scale of the problem, the existing uncertainty regarding benefit and the ethical imperative to evaluate therapies already in widespread use, there is a clear need for well-designed randomized trials.

We are therefore conducting a large, multi-country randomized platform trial to determine whether NAC reduces progression of liver injury and improves survival in patients with dengue and elevated transaminases. Patients hospitalized with moderate or severe dengue will be eligible for randomization to receive NAC versus standard of care if AST or ALT are >400 IU/L. This approach is designed to target a putative therapeutic window during the critical phase of illness when liver injury is still evolving—a stage at which interventions aimed at reducing oxidative stress and modulating inflammatory pathways may be most effective. The trial will employ partial factorial randomization, enabling evaluation of NAC both as a standalone intervention and in combination with other host-directed immunomodulators (eg, corticosteroids and JAK inhibitors). We anticipate that the trial will definitively establish whether there is a role for NAC in attenuating dengue-associated liver injury and thereby directly inform clinical practice and global policy.

## References

[1] Asish PR, Dasgupta S, Rachel G, Bagepally BS, Girish Kumar CP. Global prevalence of asymptomatic dengue infections—a systematic review and meta-analysis. Int J Infect Dis 2023; 134:292–8. 37463631 10.1016/j.ijid.2023.07.010

[2] Trung DT, Thao LTT, Hien TT, et al Liver involvement associated with dengue infection in adults in Vietnam. Am J Trop Med Hyg 2010; 83:774–80. 20889864 10.4269/ajtmh.2010.10-0090 PMC2946741

[3] Lam PK, Tam DTH, Diet TV, et al Clinical characteristics of dengue shock syndrome in Vietnamese children: a 10-year prospective study in a single hospital. Clin Infect Dis 2013; 57:1577–86. 24046311 10.1093/cid/cit594 PMC3814826

[4] Tam DTH, Ngoc TV, Tien NTH, et al Effects of short-course oral corticosteroid therapy in early dengue infection in Vietnamese patients: a randomized, placebo-controlled trial. Clin Infect Dis 2012; 55:1216–24. 22865871 10.1093/cid/cis655 PMC3466094

[5] Jaenisch T, Tam DTH, Kieu NTT, et al Clinical evaluation of dengue and identification of risk factors for severe disease: protocol for a multicentre study in 8 countries. BMC Infect Dis 2016; 16:120. 26968374 10.1186/s12879-016-1440-3 PMC4788847

[6] Yacoub S, Lam PK, Vu LHM, et al Association of microvascular function and endothelial biomarkers with clinical outcome in dengue: an observational study. J Infect Dis 2016; 214:697–706. 27230099 10.1093/infdis/jiw220 PMC4978369

[7] Wongtrakul W, Charatcharoenwitthaya K, Karaketklang K, Charatcharoenwitthaya P. Incidence of acute liver failure and its associated mortality in patients with dengue infection: a systematic review and meta-analysis. J Infect Public Health 2024; 17:102497. 39024894 10.1016/j.jiph.2024.102497

[8] Fernando S, Wijewickrama A, Gomes L, et al Patterns and causes of liver involvement in acute dengue infection. BMC Infect Dis 2016; 16:319. 27391896 10.1186/s12879-016-1656-2 PMC4938910

[9] Aye KS, Charngkaew K, Win N, et al Pathologic highlights of dengue hemorrhagic fever in 13 autopsy cases from Myanmar. Hum Pathol 2014; 45:1221–33. 24767772 10.1016/j.humpath.2014.01.022

[10] Gil L, Martínez G, Tápanes R, et al Oxidative stress in adult dengue patients. Am J Trop Med Hyg 2004; 71:652–7. 15569800

[11] Sreekanth GP, Panaampon J, Suttitheptumrong A, et al Drug repurposing of N-acetyl cysteine as antiviral against dengue virus infection. Antiviral Res 2019; 166:42–55. 30928439 10.1016/j.antiviral.2019.03.011

[12] Tenório MCDS, Graciliano NG, Moura FA, de Oliveira ACM, Goulart MOF. N-acetylcysteine (Nac): impacts on human health. Antioxidants 2021; 10:967. 34208683 10.3390/antiox10060967 PMC8234027

[13] Tian Y, Jiang W, Gao N, et al Inhibitory effects of glutathione on dengue virus production. Biochem Biophys Res Commun 2010; 397:420–4. 20510876 10.1016/j.bbrc.2010.05.108

[14] Chertoff J . N-acetylcysteine's role in sepsis and potential benefit in patients with microcirculatory derangements. J Intensive Care Med 2017; 33:87–96. 28299952 10.1177/0885066617696850

[15] World Health Organisation . WHO guidelines for clinical management of arboviral diseases: dengue, chikungunya, Zika and yellow fever. 2025. Available at: https://www.who.int/publications/i/item/9789240111110. Accessed 7 August 2025. 40690567

[16] Vasikasin V, Rojdumrongrattana T, Chuerboonchai W, et al Effect of standard dose paracetamol versus placebo as antipyretic therapy on liver injury in adult dengue infection: a multicentre randomised controlled trial. Lancet Glob Health 2019; 7:e664–70. 31000133 10.1016/S2214-109X(19)30032-4

[17] Sriphongphankul H, Liabsuetrakul T, Osatakul S. Clinical outcomes of children diagnosed dengue-associated acute liver failure with or without N-acetylcysteine treatment: a retrospective cohort study. J Trop Pediatr 2021; 67:fmab039. 34100091 10.1093/tropej/fmab039

[18] Laoprasopwattana K, Khantee P, Saelim K, Geater A. Mortality rates of severe dengue viral infection before and after implementation of a revised guideline for severe dengue. Pediatr Infect Dis J 2022; 41:211–6. 34840312 10.1097/INF.0000000000003411

[19] Dissanayake DMDIB, Gunaratne WMSN, Kumarihamy KWMPP, Kularatne SAM, Kumarasiri PVR. Use of intravenous N-acetylcysteine in acute severe hepatitis due to severe dengue infection: a case series. BMC Infect Dis 2021; 21:978. 34544380 10.1186/s12879-021-06681-9 PMC8454086

[20] Kumarasena RS, Mananjala Senanayake S, Sivaraman K, et al Intravenous N-acetylcysteine in dengue-associated acute liver failure. Hepatol Int 2010; 4:533–4. 20827413 10.1007/s12072-010-9176-4 PMC2900558

[21] Jerome RN, Zahn LA, Abner JJ, et al Repurposing N-acetylcysteine for management of non-acetaminophen induced acute liver failure: an evidence scan from a global health perspective. Transl Gastroenterol Hepatol 2024; 9:2. 38317753 10.21037/tgh-23-40 PMC10838616

[22] WHO Expert Committee on Selection and Use of Essential Medicines . The Selection and Use of Essential Medicines Report of the WHO Expert Committee on Selection and Use of EssentialMedicines, 2021 [Internet]. World Health Organization; 2022 Jan [cited 2025 Jan 28]. Available from: https://iris.who.int/bitstream/handle/10665/351172/9789240041134-eng.pdf?sequence=1.

